# *StMAPKK5* Positively Regulates Response to Drought and Salt Stress in Potato

**DOI:** 10.3390/ijms25073662

**Published:** 2024-03-25

**Authors:** Yu Luo, Kaitong Wang, Liping Zhu, Ning Zhang, Huaijun Si

**Affiliations:** 1 State Key Laboratory of Aridland Crop Science, Gansu Agricultural University, Lanzhou 730070, China; 17393144478@163.com (Y.L.); kait_wang@163.com (K.W.); ningzh@gsau.edu.cn (N.Z.); 2 College of Life Science and Technology, Gansu Agricultural University, Lanzhou 730070, China; 18893794768@163.com; 3 College of Agronomy, Gansu Agricultural University, Lanzhou 730070, China

**Keywords:** *Solanum tuberosum*, *StMAPKK5*, drought stress, NaCl stress

## Abstract

MAPKKs, as one of the main members of the mitogen-activated protein kinase (MAPK) cascade pathway, are located in the middle of the cascade and are involved in many physiological processes of plant growth and development, as well as stress tolerance. Previous studies have found that *StMAPKK5* is responsive to drought and salt stress. To further investigate the function and regulatory mechanism of *StMAPKK5* in potato stress response, potato variety ‘Atlantic’ was subjected to drought and NaCl treatments, and the expression of the *StMAPKK5* gene was detected by qRT-PCR. *StMAPKK5* overexpression and RNA interference-mediated *StMAPKK5* knockdown potato plants were constructed. The relative water content, superoxide dismutase (SOD), catalase (CAT), and peroxidase (POD) activities, as well as proline (Pro) and malondialdehyde (MDA) contents of plant leaves, were also assayed under drought and NaCl stress. The StMAPKK5 interacting proteins were identified and validated by yeast two-hybrid (Y2H) and bimolecular fluorescence complementation (BiFC). The results showed that the expression of *StMAPKK5* was significantly up-regulated under drought and NaCl stress conditions. The StMAPKK5 protein was localized in the nucleus, cytoplasm, and cell membrane. The expression of *StMAPKK5* affected the relative water content, the enzymatic activities of SOD, CAT, and POD, and the proline and MDA contents of potatoes under drought and salt stress conditions. These results suggest that *StMAPKK5* plays a significant role in regulating drought and salt tolerance in potato crop. Yeast two-hybrid (Y2H) screening identified four interacting proteins: StMYB19, StZFP8, StPUB-like, and StSKIP19. BiFC confirmed the authenticity of the interactions. These findings suggest that *StMAPKK5* is crucial for potato growth, development, and response to adversity.

## 1. Introduction

Potato (*Solanum tuberosum* L.) is the world’s fourth-largest food crop after wheat, rice, and maize. It is native to the Andes Mountains in South America and is now widely cultivated around the world [1]. Potato is also the fourth-largest staple crop in China, and it is often affected by a variety of adversities during its growth and development, with drought and soil salinization being the main environmental factors restricting its yield and quality in the northern region of China [2]. Therefore, the cloning of resistance-related genes and the mining of resistance pathways are conducive to improving potato resistance and adaptability, which is of great theoretical and practical significance to potato production.

Abiotic stresses (drought, cold, heat, osmotic stress, and salt stress) globally affect potato growth and development, resulting in reduced potato yield and quality [3]. Among them, drought and salt stresses are difficult to clearly distinguish [4]. Therefore, improving crop stress tolerance is important to ensure agricultural productivity. Mitogen-activated protein kinase (MAPK) is a class of highly conserved serine/threonine (Ser/Thr) protein kinases, which are widely found in yeast, plants, animals, and other eukaryotes, and play important regulatory roles in plant growth and development and abiotic stress tolerance [5,6]. MAPK cascade signaling pathway consists of three parts, including MAP3Ks/MAPKKKs/MEKKs/MEKKs/MKKKs, MAP2Ks/MAPKKs/MEKKs/MKKs, and MAPKs/MPKs [7,8]. Among them, MAPKKs have the fewest number of members, and to date, 83 *MAPKKKs*, 5 *MAPKKs*, and 15 *MAPKKs* have been identified from the whole potato genome [9]. The MAPK cascade is a crucial signaling module downstream of receptors and sensors that not only senses endogenous and exogenous stimuli [10,11], but also amplifies and transmits the exogenous signals step by step through phosphorylation sequential activation to participate in plant growth and development, stress tolerance, and generate specific physiological and biochemical responses in response to adverse environmental changes [12,13]. A large number of studies have shown that MAPKK plays an important role in plant growth, development, and response to abiotic stresses.

The potato *StMAPKK5* gene sequence was aligned in the *Arabidopsis* database (https://www.arabidopsis.org/index.jsp accessed on 13 January 2024), and *StMAPKK5* was found to have the highest homology with *AtMAPKK3*. When plants are subjected to adverse stress conditions, the dynamic balance of antioxidant protective enzyme systems in the body becomes unbalanced. Water deficits and high salt can disrupt this balance, leading to excessive accumulation of free radicals and reduced SOD, POD, and CAT activities, resulting in cellular damage [14]. Proline has been recognized as a unique low molecular weight osmotic agent that responds to drought and salt stress in a wide range of plant species and limits proline accumulation [15]. MDA is also one of the important products of membrane lipid peroxidation and can be used as the main indicator of membrane lipid peroxidation [16]. Activation of AtMAPKK3 by phosphorylation of AtMAPKKK18 can enhance drought tolerance in *Arabidopsis* [17]. Regarding salt stress, it has been demonstrated that the MKK3-MPK6 cascade activates *MYC2* (bHLH-like transcription factor), which regulates salt stress resistance by modulating proline biosynthesis [18]. *AtMAPKK3* was involved in drought and salt stress responses in *Arabidopsis* by phosphorylating downstream genes which enhanced drought and salt tolerance of the plant. *StMAPKK5* may also be involved in drought and salt stress responses. Reactive oxygen species (ROS) are produced by plants and stimulated by abiotic factors such as salt, low temperature, and drought lead to the activation of MEKK1-MKK4/5-MPK3/6 in response to adverse stress conditions [19]. Tomato (*Solanum lycopersicum*) *SlMKK2/5* [20], rice (*Oryza sativa*) *OsMKK1* [21,22], land cotton (*Gossypium hirsutum*) *GhMKK1/5* [23,24], and Chinese wolfberry (*Lycium chinense*) *LcMKK,* all in MKK genes’ family, respond positively to drought and salt stress [25]. However, the role of *StMAPKK5* in drought and salt stress is still not fully understood. Therefore, we analyzed the gene expression and expression pattern of transgenic plants under drought and salt stress by cloning the potato *StMAPKK5* (XM_006351467.2) gene, using subcellular localization analysis, yeast two-hybrid technology (Y2H), and bimolecular fluorescence complementation (BiFC) technology to screen and verify the reliability of proteins interacting with StMAPKK5 and to preliminarily elucidate the mechanism of *StMAPKK5* in response to drought and salt stresses in potato crop. The results of this study provide a theoretical basis for further research on the signaling pathways and biological functions of potato *StMAPKK5*. It is expected to provide a reference basis for the study of the function and regulatory mechanism of MAPKK.

## 2. Results

### 2.1. Analysis of Tissue Specificity and Expression Pattern of StMAPKK5 in Potato

**Figure 1:**
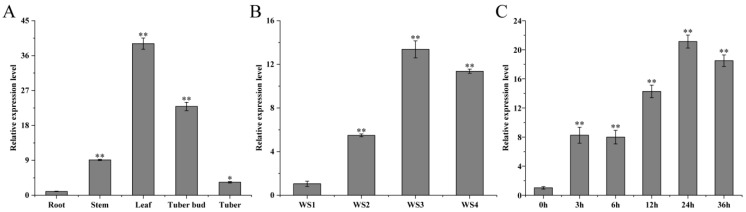
Tissue specificity and expression pattern analysis of potato *StMAPKK5*. (**A**) The relative expression level of *StMAPKK5* in different organs of potato. (**B**) Relative expression of the *StMAPKK5* gene under drought stress. WS1: water stress group 1; WS2: water stress group 2; WS3: water stress group 3; WS4: water stress group 4. (**C**) Changes of potato *StMAPKK5* gene expression under NaCl stress conditions. qRT-PCR determined relative expression levels expressed as 2^−ΔΔCt^, relative to *StEF1α* gene expression. Each column represents the mean values ± SE (*n* = 3; * *p* < 0.05; ** *p* < 0.01).

The qRT-PCR results showed that the expression levels of *StMAPKK5* in roots, stems, leaves, tuber buds, and tubers of the potato variety ‘Atlantic’ were significantly different (* *p* < 0.05). The relative expression levels were highest in leaves and lowest in tubers, and were 38.91 and 3.34 times higher than those in roots for leaves and tubers, respectively, and 15.24 and 22.80 times higher than those in roots for stems and shoots, respectively (Figure 1A). To explore the possible role of *StMAPKK5* in adversity signaling, the expression level of *StMAPKK5* was examined under drought and NaCl stress using qRT-PCR. The results showed that the expression level of this gene in leaves showed a tendency to increase and then decrease at a relative soil water content of WS1 (75~85%) as a control, and the highest expression of *StMAPKK5* was found at a soil water content of WS3 (35~45%), which was 13.38-fold higher than that of the control. The lowest expression level of *StMAPKK5* was found at soil water content of WS2 (55~65%), which was 5.49 times higher than that of the control group (Figure 1B). Under the treatment of NaCl, the expression of *StMAPKK5* showed a fluctuating trend and reached a peak at 24 h, which was 20.60-fold then that at 0 h. The expression of *StMAPKK5* was also up-regulated under the treatment of droughts and NaCl. The results showed that both droughts and NaCl treatments induced *StMAPKK5* to up-regulate its expression, indicating that the expression pattern of *StMAPKK5* was different under different treatments (Figure 1C).

### 2.2. Subcellular Localization Assay

**Figure 2:**
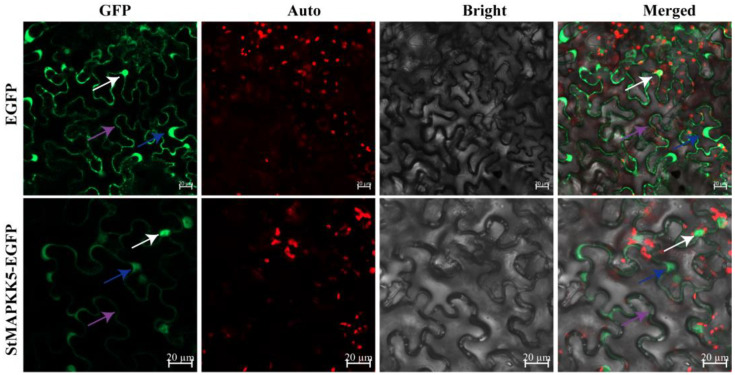
Subcellular localization of StMAPKK5. The EGFP and StMAPKK5-EGFP fusion protein transiently expressed in tobacco; white, blue, and purple arrows indicate the nucleus, cytoplasm, and cytoplasmic membrane; GFP: EGFP fluorescence signal in the dark field: Auto: auto-fluorescence of chloroplast; Bright: cell morphology under bright field; Merged: combination field. (bar = 20 μm).

StMAPKK5 was predicted to be mainly localized in the nucleus and cytoplasm through the online website PSORT Prediction, to further verify the reliability of the prediction results. The pCAMBIA1300-35S-EGFP empty vector was used as a negative control, and the StMAPKK5-EGFP fusion protein was injected into tobacco leaves by *Agrobacterium*-mediated transformation, and the expression of the fusion protein was observed under a laser confocal scanning microscope. The results showed that the StMAPKK5-EGFP fusion protein had strong GFP green fluorescence in the nucleus, cytoplasm, and cytoplasmic membrane, and the control had EGFP green fluorescence signals in the nucleus, cytoplasm, and cytoplasmic membrane (Figure 2), which indicated that the StMAPKK5 protein was localized in the nucleus, cytoplasm, and cytoplasmic membrane.

### 2.3. Genetic Transformation of StMAPKK5 and Identification of Transgenic Potato Plants

**Figure 3:**
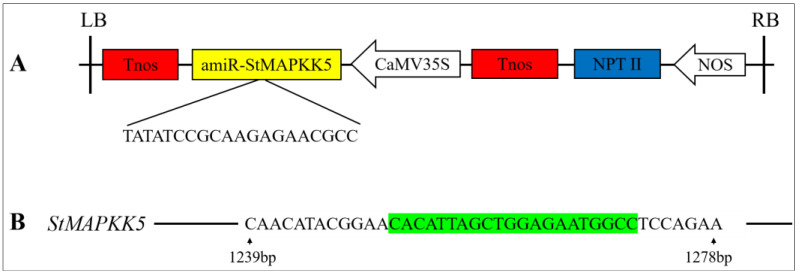
RNAi-mediated gene silencing in transgenic potato plants. (**A**) Schematic illustration of the engineered pCPB121-amiR-StMAPKK5 vector. Tnos: Terminator; amiR-StMAPKK5: Artificial microRNAs (amiRNAs); CaMV35S: CaMV 35S promoter; *NPT* II: Expression of a reporter gene (Neomycin phosphotransferase II gene) on vector pCPB121; NOS: promoter. (**B**) Schematic illustration of the target region of the *StMAPKK5* gene. Green part of the sequence: miRNA repressor sequence of *StMAPKK5*.

**Figure 4:**
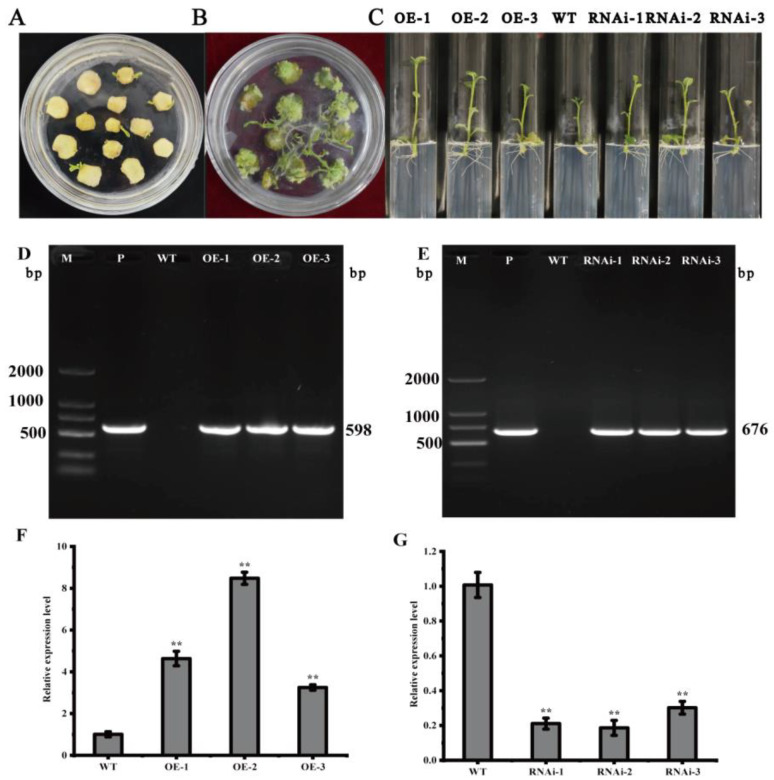
Acquisition and characterization of transgenic plants. (**A**,**B**) Calli and differentiated buds. (**C**) Rooting and screening of transgenic plants. (**D**,**E**) PCR assay of transgenic plants. (**F**,**G**) Relative expression of *StMAPKK5* in transgenic and WT plants. M: DL 2000 marker; P: Positive control plasmid; WT: ‘Atlantic’ wild-type plants; OE-1~OE-3: plants carrying recombinant plasmid pCAMBIA1300-35S-StMAPKK5; RNAi-1~RNAi-3: ‘Atlantic’ transgenic plants carrying recombinant plasmid pCPB121-amiR-StMAPKK5. Each column represents the mean ± SE (*n* = 3; ** *p* < 0.01).

In vitro infection of the potato variety ‘Atlantic’ microtubers using *Agrobacterium* solution contained the overexpression vector pCAMBIA1300-35S-StMAPKK5 and the down-expression vector pCPB121-amiR-StMAPKK5 (Figure 3) and formed calli and differentiated buds on the differentiation medium. Then, the roots were sieved using a medium containing hygromycin and kanamycin (Figure 4A,B). Rooted transgenic plants were screened by a rooting medium containing hygromycin and kanamycin, respectively (Figure 4C). The successfully transformed plants were identified by amplification of the reporter gene *HYG* in the overexpression vector pCAMBIA1300-35S-StMAPKK5 and the reporter gene *NPT* II in the down-expression vector pCPB121-amiR-StMAPKK5, respectively. *HYG* gene could be amplified to 598 bp from the transgenic plants of the overexpression vector (Figure 4D), and the *NPT* II gene could be amplified to 676 bp from the transgenic plants of the down-expression vector (Figure 4E), and two reporter genes could not be amplified from the WT plants, which indicated that the transgenic plants were successfully obtained (Figure 4F,G). The relative expression levels of *StMAPKK5* in WT plants and transgenic plants were analyzed by qRT-PCR (Figure 4F,G). The results showed that the relative expression of *StMAPKK5* in transgenic plants OE-1, OE-2, and OE-3 was 4.63-fold, 8.47-fold, and 3.25-fold higher than that in WT plants, respectively, and it was significantly higher than that in WT plants. The relative expression of RNAi-1, RNAi-2, and RNAi-3 was 0.21-fold, 0.19-fold, and 0.30-fold higher than that in WT plants, respectively, and they were significantly lower than those of WT plants. These results demonstrated that overexpressed and down-regulated transgenic plants had been successfully obtained.

### 2.4. StMAPKK5 Positively Regulates Drought Resistance and Salt Tolerance of Potato

**Figure 5:**
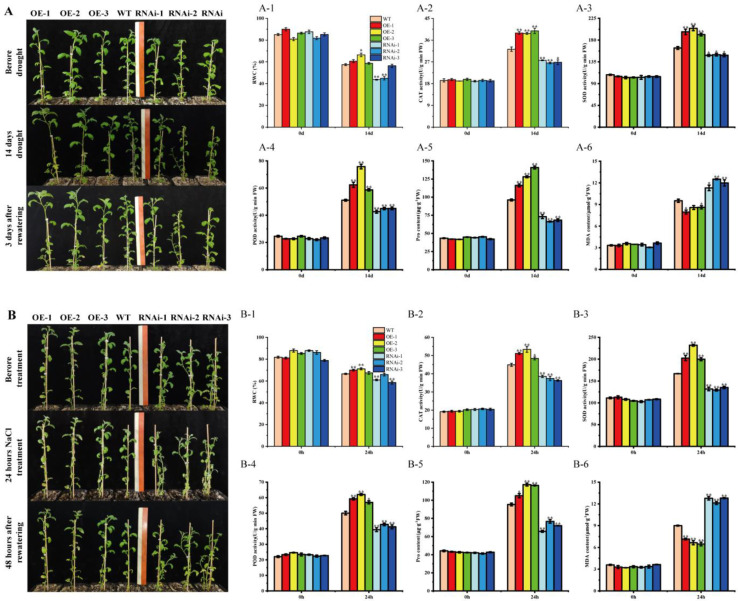
Physiological and biochemical indices of transgenic plants under natural drought. (**A**) Phenotypes of transgenic potatoes after 14 d of drought stress and 3 d of rehydration treatment; Scale bars = 30 cm. (**B**) Phenotypes of transgenic potatoes after 24 h of NaCl stress and 48 h of rehydration treatment. (**A-1**,**B-1**) Relative water content of leaves. (**A-2**,**B-2**) CAT activity. (**A-3**,**B-3**) SOD activity. (**A-4**,**B-4**) POD activity. (**A-5**,**B-5**) proline content. (**A-6**,**B-6**) MDA content. Each column represents the mean ± SE (*n* = 3; * *p* < 0.05; ** *p* < 0.01).

To further investigate whether *StMAPKK5* overexpression or RNAi interference-expressing plants affect potato tolerance to drought and salt stress, 6-week-old wild-type plants (WT), overexpression plants (OE-n), and RNAi interference-expressing plants (RNAi-n) with basically the same growth conditions were selected for natural drought and NaCl stress treatment, respectively. It was observed that the growth condition of transgenic *StMAPKK5* OE-n grew significantly better than the WT, while the opposite was true for the RNAi-n, and the leaves of RNAi-n lines were obviously wilting (Figure 5A,B). The relative water content of plant leaves reflected the drought tolerance of plants to some extent. After drought treatment, the water content of the leaves of OE-n plants was higher than that of WT plants, while that of RNAi-n plants was lower than that of WT plants, and the results were same as those after drought and NaCl treatment (Figure 5A-1,B-1).

**Figure 6:**
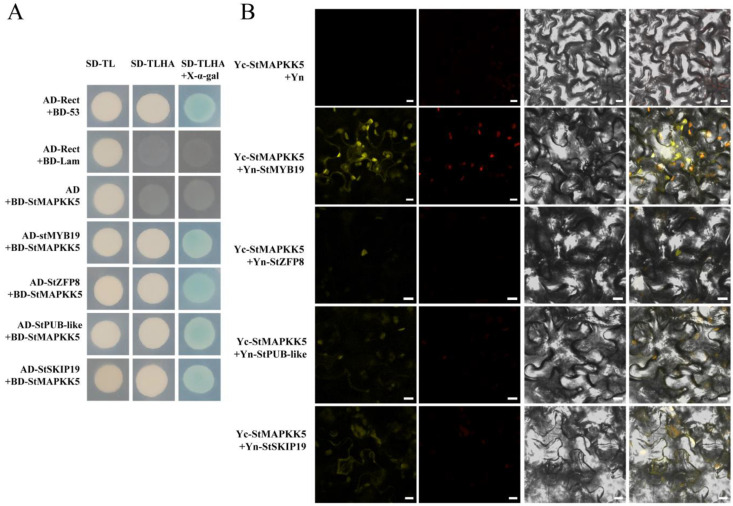
(**A**) Y2H and BiFC validation of the interaction between StMAPKK5 protein and interacting proteins. SD-TL represents -Trp -Leu dystrophic medium; SD-TLHA represents -Trp-Leu -His-Ade dystrophic medium to which x-α-gal was added to promote blue color. Positive control: AD-Rect+BD-53; Negative control: AD-Rect + BD-Lam, and the rest are experimental groups. (**B**) BiFC analysis to detect StMAPKK5 interaction in tobacco. Yc-StMAPKK5 + Yn-StMYB19/StZFP8/StPUB-like/StSKIP19 was used as the experimental group; Yc-StMAPKK5 + Yn was the negative control; yellow color was YFP fluorescence. (bar = 10 μm).

Subsequently, the activities of three key antioxidant enzymes, CAT, SOD, and POD, as well as the proline and MDA contents, were determined. The results showed that CAT, SOD, and POD activities of WT, OE-n, and RNAi-n plants did not differ significantly before the treatments, and after drought and NaCl treatments, CAT, SOD, and POD activities of OE-n plants were significantly higher than those of WT and RNAi-n plants, and WT plants were significantly higher than those of RNAi-n plants (Figure 5A-2–A-4,B-2–B-4). Drought-induced accumulation of proline and MDA contents of OE-n plants were not significantly different from those of WT and RNAi-n plants before treatment. The accumulation of Proline and MDA contents in OE-n plants were significantly higher than that in WT plants after treatments. The results of RNAi-n plants were opposite of those of WT and RNAi-n plants (Figure 5A-5,B-5). The MDA content of OE-n plants was significantly lower than that of WT and RNAi-n plants, and RNAi-n plants were higher than that of WT plants (Figure 5A-6,B-6).

### 2.5. Yeast Two-Hybridization to Identify StMAPKK5-Interacting Proteins

To further investigate the function of StMAPKK5 in the drought and salt stress response of potato, this study screened a variety of proteins interacting with StMAPKK5 involving different physiological and biochemical processes using Y2H. We mainly analyzed the following four proteins, StMYB19 (NM_001318681), StZFP8 (XM_006362556.1), StPUB-like (XM_015304533.1) and StSKIP19 (XM_015303010.1), which may be involved in drought and salt-responsive regulation in plants. Yeast cells co-transformed by pGADT7-StMYB19, pGADT7-StZFP8, pGADT7-StPUB-like, and pGADT7-StSKIP19 with pGBKT7-StMAPKK5 and cells were transformed by the positive control plasmid, which was grown on SD/Ade/-His/-Leu/-Trp/X-α-gal media, respectively. The yeast of each combination was grown normally on the medium SD/Ade/-His/-Leu/-Trp/X-α-gal, and the colonies of the positive control group and the experimental group turned blue (Figure 6A). This indicated that the reporter β-galactosidase gene Z (LacZ) was activated and expressed in a functional β-galactosidase, suggesting that the StMAPKK5 interacted with StMYB19, StZFP8, StPUB-like, and StSKIP19, respectively. To further verify the results of Y2H experiments, BiFC experiments were performed and YFP was used as a marker in *Nicotiana benthamiana* to characterize the interaction of StMAPKK5 with the interacting protein. The interacting proteins were transiently co-expressed by ligating the interacting proteins to pSPYNE-35S and StMAPKK5 to pSPYCE-35S, respectively, in equal proportions and mixed and injected into the leaves of *N. benthamiana* leaves. The YFP fluorescent signal was found to be expressed in both the cell membrane and nucleus, whereas the signal in the negative control was not detected (Figure 6B). These results suggested that the StMAPKK5 interacted with StMYB19, StZFP8, StPUB-like, and StSKIP19, respectively.

**Figure 7:**
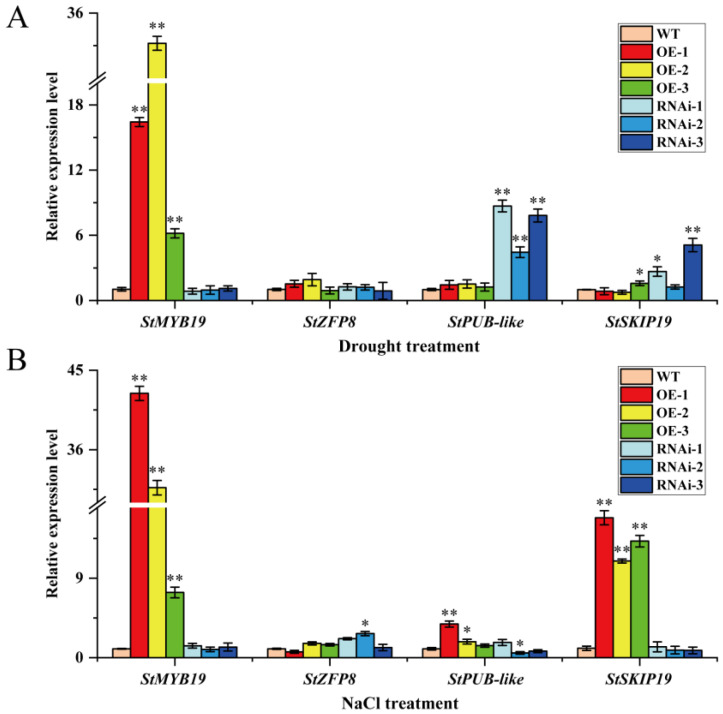
Relative expression of *StMYB19*, *StZFP8*, *StPUB-like*, and *StSKIP19* genes in leaves of wild-type and transgenic *StMAPKK5* plants detected by qRT-PCR. qRT-PCR determined relative expression levels expressed as 2^−ΔΔCt^, relative to *StEF1α* gene expression. (**A**) Relative expression of *StMYB19*, *StZFP8*, *StPUB-like* and *StSKIP19* genes under drought stress. (**B**) Relative expression of *StMYB19*, *StZFP8*, *StPUB-like* and *StSKIP19* genes under NaCl stress. Each column represents the mean values ± SE (*n* = 3; * *p* < 0.05; ** *p* < 0.01).

To further explore the possible mechanism of StMAPKK5 and the four interacting proteins in adverse stress conditions, the target product was amplified by PCR (Supplementary Figure S3), and the primer sequences are shown in Supplementary Table S2. The relative expression of *StMYB19*, *StZFP8*, *StPUB-like* and *StSKIP19* genes (Gene CDS sequences are shown in Supplementary Table S1) was detected by qRT-PCR in the leaves of WT, OE-n, and RNAi-n plants under drought and NaCl treatments. The results showed that the gene expression of *StMYB19* in OE-n plants was significantly higher than that in WT and RNAi-n plants (Figure 7A), under drought treatments. The *StMYB19* may have synergized with *StMAPKKK5* to positively regulate the drought, as there was not much difference in the expression of *StZFP8* in WT, OE-n, and RNAi-n plants (Figure 7A), and *StZFP8* played a regulatory role in other metabolic or signal transduction pathways. The gene expression of *StPUB-like* in RNAi-n plants was significantly higher than that in WT and OE-n plants (Figure 7A), while *StPUB-like* was phosphorylated in the MAPK signaling pathway with no expression. The expression of the *StSKIP19* gene was slightly higher in RNAi-n plants than in OE-n and WT plants (Figure 7A), whereas *StSKIP19* synergized with *StMAPKK5* to negatively regulate the drought tolerance. Under NaCl treatment, the results showed that the gene expression of *StMYB19* in OE-n plants was significantly higher than that in WT and RNAi-n plants, while *StMYB19* synergized with *StMAPKK5* to positively regulate salt stress (Figure 7B). The expression of *StZFP8* and *StPUB-like* genes did not differ very much in WT, OE-n, and RNAi-n plants (Figure 7B). The *StZFP8* and *StPUB-like* genes played a regulatory role in other metabolic or signaling pathways. The expression of the *StSKIP19* gene in OE-n plants was significantly higher than that in WT and RNAi-n plants (Figure 7A), whereas *StSKIP19* was synergistically positively regulated by NaCl stress with *StMAPKK5*.

## 3. Discussion

Potato is a significant food crop around the world. Drought and salt adversity are two important environmental factors that affect its yield and quality [26]. In recent years, the role of MAPK in plant stress tolerance has attracted much academic attention. MAPKKs play important functions in drought and salt stress responses in plants. Song et al. [27] found that the watermelon *ClMAPKK3/5* showed up-regulated expression of varying degrees when subjected to abiotic stress, but drought and salt stresses inhibited its significantly down-regulated expression, suggesting that this gene plays an important regulatory function in adversity stress. AtMAPKK3, which is homologous to potato StMAPKK5, regulates the enhancement of drought tolerance in *Arabidopsis* through the typical ABA signaling pathway activated by *AtMAPKKK18* phosphorylation [28]. It can be seen that the MAPKs cascade pathway plays an important role in responding to abiotic stresses. The *StMAPKK5* coding region is 1548 bp in length and the encoded protein contains 515 amino acid residues. It was analyzed with a NCBI conserved structural domain score and predicted to have a serine/threonine protein kinase (Pkinase) structural domain and to be localized at 80-366 aa (Supplementary Figure S1). By analyzing the NCBI Splign, the *StMAPKK5* was found to contain eight introns and nine exons, which is a discontinuous gene (Supplementary Figure S1). There were 51 phosphorylation sites in a total score of 0.5–1, accounting for 9.90% (Supplementary Figure S1). This study focused on the role of *StMAPKK5* in the response to drought and salt stress in potatoes and analyzed its regulatory mechanisms. The results showed that *StMAPKK5* was expressed in all tissues, but highest in leaves and lowest in roots (Figure 1). The highest expression of *StMAPKK5* was found at a soil water content of WS2 (55~65%) (Figure 1). Potato plants were treated with NaCl, and then qRT-PCR was performed to analyze the expression of *StMAPKK5*. *StMAPKK5* was significantly up-regulated under NaCl treatments and its expression peaked at 24 h of the stress treatment (Figure 1). Therefore, we hypothesized that StMAPKK5-related expression might improve the stress tolerance of potato plants. The results of subcellular localization of StMAPKK5 showed that its fusion protein was localized in the nucleus, cytoplasm, and cell membrane (Figure 2), which was consistent with the observation of potato StMAPK3 and maize ZmMAPKK3 [29,30]. In previous studies related to MAPKK localization, most MAPKK proteins were localized in the cytoplasm; however, localization in the nucleus was also reported [27], which may be because MAPKK is phosphorylated in the cytoplasm by the upstream of MAPKKK and then transferred into the nucleus by phosphorylation of its downstream of MAPKK, resulting from the phosphorylation of MAPKK in the cytoplasm.

To further investigate the function of *StMAPKK5*, WT and transgenic StMAPKK5 plants were bred and treated with drought and NaCl stress, respectively. It is hypothesized that the change of *StMAPKK5* expression may be involved in the mechanism of drought and salt tolerance in plants to some extent. RWC is an important parameter for drought stress in plant science, and adequate water status can represent plant tolerance to drought and salt stresses [31,32]. Many studies have focused on plant growth and antioxidant enzyme systems in stress resistance [33]. Due to the influence of adversity (drought, high temperature, salinity, and low temperature, etc.), the antioxidant protective enzyme system in the body will be imbalanced, which damages the membrane lipid structure, causing damage to the plant cellular processes. Protective enzymes system such as SOD, POD, and CAT are effective in breaking down free radicals and maintaining reactive oxygen species at a low level in the organism. Water stress and high salt can disrupt this balance, leading to excessive accumulation of free radicals and reduced SOD, POD, and CAT activities, resulting in cellular damage [14]. In our study, drought induced the accumulation of proline, CAT, SOD, and POD activities. The outcomes showed that the activities of the antioxidant enzyme system in OE-n plants were significantly higher than those in WT under drought and NaCl stress, while the opposite results were observed in RNAi-n plants, which indicated that overexpression of the *StMAPKK5* enhanced the ability to scavenge reactive oxygen species and attenuated the corresponding oxidative damage, thus increasing the resistance of potato plants to drought and salt stress (Figure 5). MDA is one of the imperative products of membrane lipid peroxidation that can be used as one of the main indicators of membrane lipid peroxidation [34]. The MDA content in OE-n plants was significantly lower than that of WT, whereas in RNAi-n lines, it was higher than that of WT strains (Figure 5). This indicated that the *StMAPKK5* reduced the malondialdehyde content in potato plants under drought and NaCl stress, thereby reducing the lipid peroxidation damage to the cell membranes of potato plants under drought and salt stress, and making the potato plants resistant to drought and salt stress. Increased accumulation of proline content under drought stress is a marker of drought tolerance, and proline accumulation in OE-n lines was significantly higher than in WT plants, whereas in RNAi-n lines, it was significantly lower than that in WT lines, suggesting that the *StMAPKK5* improved the tolerance of potato plants under drought and salt stress by increasing the content of the osmotic substances such as proline and MDA, etc. (Figure 5). In other reports, overexpression of *StMAPK11* under drought conditions resulted in higher SOD, CAT, and POD activities, increased proline content, and decreased H_2_O_2_ and MDA content, which in turn increased antioxidant activity and photosynthesis, and significantly enhanced drought tolerance of potato plants [35]. Likewise, in other species such as poplar, *PtMAPKK4* overexpression plants showed higher antioxidant enzyme activities after drought stress, which significantly improved the drought stress tolerance of transgenic poplar [36]. Overexpression of grape *VvMKK2* in *Arabidopsis thaliana* improved salt tolerance and drought resistance, while overexpression of *VvMKK4* only improved salt tolerance [37]. Our experimental results showed that *StMAPKK5* could improve the oxidative scavenging capacity of potato plants under drought and NaCl stress treatment, thus reducing the damage of drought and salt stress on potatoes. Through systematic research and analysis, the growth, physiological, and biochemical changes in potatoes under drought and salt stress were initially explored, which provides an important theoretical basis for the study of drought resistance of potatoes.

To further investigate the molecular mechanism of StMAPKK5 in drought and salt stress response in potatoes, this study verified and validated four proteins interacting with StMAPKK5 using Y2H and BiFC, and the results showed that StMAPKK5 interacts with StMYB19, StZFP8, StPUB-like, and StSKIP19 proteins (Figure 6). Subsequently, the relative expression of *StMYB19*, *StZFP8*, *StPUB-like*, and *StSKIP19* in the leaves of WT, OE-n, and RNAi-n potato plants under drought and NaCl treatment was detected by qRT-PCR (Figure 7). The results showed that the proteins interacting with *StMAPKK5* functioned in different physiological adversities. The gene expression of *StMYB19* was significantly higher in OE-n plants than in WT and RNAi-n plants under drought and NaCl treatments, and *StMYB19* may positively regulated the drought and salt stress in synergy with *StMAPKK5*. It was found that MYB regulates the development of root hairs and plays a crucial role in plant resistance to abiotic stresses (salt stress, drought stress, and high-temperature stress) [38]. In potato, the expression level of *StMYB* was measured by qRT-PCR in ABA, IAA, GA_3_, high temperature (35 °C), drought, and NaCl, and it was found that the highest expression of *StMYB19* was found under ABA treatment, and the expression of the gene was significantly increased under drought and NaCl treatments [39]. The findings of the present study are consistent with those already reported; therefore, *StMYB19* synergistically and positively regulated drought and salt stress with overexpression of *StMAPKK5* in potato crop. Under drought and NaCl treatments, there was little difference in the expression of S*tZFP8* in WT, OE-n, and RNAi-n potato plants. *StZFP* showed different expression patterns under multiple stresses, and eight of them were involved in response to abiotic stresses and might enhance plant tolerance to salt and drought stresses [40]. However, *StZFP8* has not been reported to regulate drought and salt stress in potato, but *StZFP8* played a regulatory role in other metabolic or signaling pathways. Under drought and salt stress, the gene expression of *StPUB-like* was significantly higher in RNAi-n plants than in WT and OE-n plants, and *StPUB-like* may be phosphorylated as a regulator in the MAPK signaling pathway without functioning. Under salt stress, there was little difference in the expression of *StPUB-like* in WT, OE-n, and RNAi-n potato plants, and *StZFP8* played a role in other metabolic or signaling pathways as a salt-responsive mechanism. The ubiquitination pathway is widely involved in the regulation of plant growth, development, and stress response. U-box proteins play important roles in the plant ubiquitination pathway through their E3 ubiquitin ligase activity. In recent years, *Arabidopsis* ARM/U-box proteins have been found to have important roles in response to plant growth, development, and response to environmental stresses [41]. In *Arabidopsis*, the complex formed by PUB19 with UBC32/33/34 inhibits abscisic acid-mediated stomatal closure and drought stress tolerance and is a negative regulator of ABA signaling [42]. The U-box E3 ligases PUB18/PUB19 and PUB22/PUB23 are negative regulators in response to drought stress [43,44,45]. In potatoes, StPUB27 can respond to drought stress by regulating stomatal conductance [46]. Co-expression of the StUBC18-StPUB40 gene pair leads to diminished potato ROS scavenging under drought stress, thereby negatively regulating potato tolerance to drought stress [41]. MEKK1 ubiquitination functions by inhibiting MEKK1-catalyzed phosphorylation of MKK1 and MKK4, leading to inhibition of ERK1/2 and JNK activation, which is used to control MAPK kinase activity and MAPK signaling mechanisms in cells [47]. *MAPKKKK18* and *MAPKKK17* act as the downstream module; MKK3-MPK1/2/7/14 arbitrates ABA-mediated responses and is regulated by the E3 ligase that plays a central role in ABA signaling, drought tolerance, and senescence [48]. Under drought stress, the expression of *StSKIP19* in WT, OE-n, and RNAi-n potato plants did not differ significantly, and *StSKIP19* played a chief role in other metabolic or signaling pathways, such as drought response mechanisms. Under NaCl treatment, the gene expression of *StSKIP19* was significantly higher in OE-n plants than in WT and RNAi-n plants, and *StSKIP19* synergized with *StMAPKK5* to positively regulate drought. *SKIP*, an essential factor for normal plant growth and development, is widespread and highly conserved in higher organisms from yeast to humans, and acts as a transcriptional cofactor in abiotic stresses. In rice, Hou et al. [49] proposed that *OsSKIP* mediates stress responses by regulating many stress-related genes at the transcriptional level. Under salt stress conditions, *SKIP* is required for precise selective splicing of genes, including salt tolerance genes. The mRNA splicing machinery in *Arabidopsis* contributes to salt response at the post-transcriptional level, and *SKIP* provides a link between selective splicing and salt tolerance. However, the responsive mechanisms of StMYB19, StZFP8, StPUB-like, and StSKIP19 under drought and salt tolerance in potato crop are not well understood, and further experiments are needed to demonstrate the possible mechanisms of these pathways.

## 4. Materials and Methods

### 4.1. Growth Conditions and Treatment of Plant Materials

Stem segments of potato cultivar ‘Atlantic’ were inoculated in MS solid medium containing 3% and 8% sucrose, and placed in a light incubator at (22 ± 1) °C, with 16 h of light/8 h of darkness for 30 d. After 30 days, plants inoculated with 8% sucrose MS solid medium were cultured in a dark environment for 45–60 days to obtain the microtubers [50]. Potato plants inoculated with 3% sucrose MS solid medium were transplanted into potted plants of 10 cm × 10 cm (vermiculite: nutrient soil = 2:1) and continued to be cultivated for 20–30 d at a temperature of (22 ± 1) °C, a light intensity of 2000 Lx, and 16 h of light/8 h of darkness. After that, we chose the growth conditions of plants that were essentially identical for subsequent treatments. Roots, stems, leaves, tubers, and tuber buds of ‘Atlantic’ were collected for tissue-specific analysis. Drought and salt (NaCl) treatments were carried out when the plants grew to a height of about 25 cm. Soil water content was monitored using a TDR-300 sensor (Spectrum R, Aurora, IL, USA) at a fixed time each day. For the drought treatment, sampling was carried out when the relative soil moisture content reached 75% to 85% (WS1), 55% to 65% (WS2), 35% to 45% (WS3), and 15% to 25% (WS4), respectively, and a relative moisture content of 75% to 85% (WS1) was used as a control. The top 3rd to 5th leaves of the plant were collected, and three biological replicates were performed. For NaCl treatment, plants were watered with 150 mL of 200 mmol/L NaCl. When the stress time was at 0 h, 3 h, 6 h, 12 h, 24 h, and 36 h, the leaves of the plants were collected and three biological replicates were performed.

*N. benthamiana* was selected for this study. *N*. *benthamiana* has become one of the most commonly used model plant species for research on molecular plant-microbe interactions as well as other areas of plant science. *N*. *benthamiana* is native to Australia and belongs to the Solanum family (*Solanum melongena* L.), and is a heterotetraploid plant with 19 chromosomes. The estimated genome size of *N*. *benthamiana* is 3 Gb (gigabases) [51]. *N*. *benthamian* was grown in 10 cm × 10 cm pots (vermiculite: nutrient soil = 2:1) with a light intensity of 2000 Lx at (22 ± 1) °C, 16 h light/8 h dark, for about 30–45 d. All experiments were carried out in three biological replicates and three technical replicates.

### 4.2. Cloning of StMAPKK5

The expression pattern of potato *StMAPKKs* genes under abiotic stress was analyzed in the laboratory [52], and the results showed that the expression of most of the genes (except *StMAPKK3*) in MAPKKs was up-regulated under stress treatments, among which *StMAPKK5* was up-regulated under drought and salt stress. Therefore, *StMAPKK5* was selected as the target gene in this study. The *StMAPKK5* gene sequence (ID: Soltu.DM.03G023940.1) was retrieved from the potato database Spud DB (http://spuddb.uga.edu/index.shtml accessed on 13 January 2024). The total RNA extraction and cDNA first strand synthesis of potato cultivar ‘Atlantic’ was performed using the TRNzol Universal Plant Total RNA Extraction Kit and FastKing gDNA Dispelling RT SuperMix Reverse Transcription Kit (Tiangen, Beijing, China), respectively. The CDS region of the *StMAPKK5* gene was cloned from the cDNA library of leaves of the potato variety ‘Atlantic’ as a template (Gene CDS sequences are shown in Supplemental Table S1). The primer sequences are shown in Supplementary Table S2. The reaction system was PrimerSTAR HS (Premix) 12.5 μL, ddH_2_O 9 μL, cDNA template 1.5 μL, and pCAMBIA1300-35S-StMAPKK5-F/R 1 μL; the reaction conditions were: pre-denaturation: 98 °C for 3 min; denaturation: 98 °C for 10 s, annealing: 55 °C for 5 s, extension: 72 °C for 100 s, cycling 34 times, extension 72 °C for 5 min.

### 4.3. StMAPKK5 Expression Analysis by qRT-PCR

The specific expression of the *StMAPKK5* was determined by qRT-PCR concerning the instructions of the 2 × Universal Blue SYBR Green qPCR Master Mix kit (Accurate Biology, Changsha, China), and the primer sequences are shown in Supplementary Table S2. The *StEF1α* elongation factor (GenBank ID: AB061263.1) was used as a standardized reference gene, the reaction system was 1 μL cDNA (100 ng) template, 10 μL 2 × Universal Blue SYBR Green qPCR Master Mix, 0.5 μL StMAPKK5-F/R, and 8.0 μL ddH_2_O. on a Light Cycler 96 system (Roche, Diagnostics GmbH, Basel, Switzerland). qRT-PCR was performed in a Light Cycler 96 system with the following parameters: 95 s °C for 30 s followed by 40 cycles of 95 °C for 15 s, and 60 °C for 30 s. All experiments were performed in three biological replicates and three technical replicates. The relative expression of the StMAPKK5 in different tissues and time was calculated using the method of 2^−ΔΔCt^ [53].

### 4.4. Subcellular Localization of StMAPKK5

The *StMAPKK5* subcellular localization vector was constructed by homologous recombination, and the primers were designed according to the coding region of the *StMAPKK5* sequence and the sequence of the pCAMBIA1300-35S-EGFP vector using the TaKaRa online website, and the CDS sequence of *StMAPKK5* without terminator was amplified using the cDNA library of the potato variety ‘Atlantic’ as the template. The recombinant plasmid pCAMBIA1300-EGFP-StMAPKK5 was obtained by inserting the appropriate PCR product into the *Kpn* I and *Sal* I restriction site of the pCAMBIA1300-35S-EGFP vector. Then the empty vector pCAMBIA1300-35S-EGFP and the recombinant plasmid pCAMBIA1300-EGFP-StMAPKK5 were introduced into *Agrobacterium tumefaciens* GV3101(Angyubio, Shanghai, China), respectively. The tobacco infection solution was prepared by referring to the method of Qi et al. [54], and the infiltration solution was injected with a disposable sterile syringe needle from the back of the tobacco leaf (the 2nd to 4th leaf of the 5–7-week-old tobacco leaves from the top to the bottom) were injected with the infection solution from the abaxial surface and the infested area was marked. After 48–72 h of incubation at (23 ± 2) °C under dark conditions, the distribution of green fluorescent signals of pCAMBIA1300-EGFP-StMAPKK5 was observed under a laser confocal scanning electron microscope (CARI ZEISS, LSCM 800, Oberkochen, Baden-Württemberg, Germany) to determine the sites of StMAPKK5 expression in the cells.

### 4.5. Construction of Plant Expression Vectors

Specific primers were designed according to the *StMAPKK5* CDS sequence and the vector pCAMBIA1300-35S-EGFP sequence. The target product was amplified by PCR (Supplementary Figure S2), and the primer sequences are shown in Supplementary Table S2. The PCR product was linked by homologous recombination to the vector pCAMBIA1300-35S-EGFP containing *Kpn* I and *Sal* I restriction sites. The overexpression vector was named pCAMBIA1300-35S-StMAPKK5 after validation by double enzyme digestion and identification by sequencing. For downregulated expression vectors, Oligo from the online site WMD3 was used to design precursor primers (I, II, III, and IV) (Supplementary Table S2) and obtain the target fragment by standard PCR [55]. The PCR product was ligated into pMD™ 18-T vector (TaKaRa Bio, Beijing, China), and after double enzyme digestion verification and sequencing, the target fragment was ligated to the *Kpn* I and *Sac* I double digestion vector pCPB121 by T4 ligase, and the sequencing was correctly named as pCPB121-amiR-StMAPKK5. The successfully identified recombinant plasmid was transformed into *Agrobacterium tumefaciens* GV3101(Angyubio, Shanghai, China) [56].

### 4.6. Genetic Transformation of Potatoes

The genetic transformation of potato microtubers was based on the transformation method of Si et al. [57]. The potato cultivar ‘Atlantic’ was selected as the experimental material, and the microtubers were cut into slices with a thickness of 0.3–0.4 cm with a sterile blade. The slices were transferred to *Agrobacterium tumefaciens* solution containing the recombinant plasmids pCAMBIA1300-35S-StMAPKK5 and pCPB121-amiR-StMAPKK5 infected for 7–10 min, respectively, and then the remaining bacterial liquid on the slices was sucked up with a sterile dry filter paper and placed on a solid MS medium and cultured in darkness for 2 days under the condition of 28 °C. The co-cultured potato slices were transferred to a differentiation medium at 25 °C under 2500 Lx to continue incubation, and the medium was changed once a week. When the differentiated shoots grew to 1.2–1.5 cm, they were cut and transferred to a rooting medium containing kanamycin (75 mg/L) or hygromycin (50 mg/L) for at least three rooting screening [54], and initially identified as transformed plants. To identify the transgenic plants, the DNA of the transgenic plants was extracted by the CTAB method, the wild-type plants were used as the negative control, and the constructed plant expression vector plasmid was used as positive control. The hygromycin (*HYG*) gene on the overexpression vector and the neomycin phosphotransferase (*NPT* II) gene on the repressor expression vector were used for PCR detection, and the fragment sizes were 598 bp and 676 bp, respectively. The primer sequences are shown in Supplementary Table S2. Transgenic plants transformed with pCAMBIA1300-35S-StMAPKK5 and pCPB121-amiR-StMAPKK5, respectively, were named OE-n and RNAi-n. Successful transgenic plants were further characterized by PCR and qRT-PCR for subsequent studies.

### 4.7. Drought and NaCl Stress Treatment

Stem segments of potato cultivar ‘Atlantic’ were inoculated in MS solid medium containing 3% and 8% sucrose, and placed in a light incubator at (22 ± 1) °C, 16 h of light/8 h of darkness for 30 d. Potato plants inoculated with 3% sucrose MS solid medium were transplanted into potted plants of 10 cm × 10 cm (vermiculite: nutrient soil = 2:1) and continued to be cultivated for 20–30 d at a temperature of (22 ± 1) °C, a light intensity of 2000 Lx, and 16 h of light/8 h of darkness. After that, we chose the growth conditions of plants that were essentially identical for subsequent treatments. Watering was done at 3 d intervals afterward. Drought and salt stress treatments were performed when plants grew to a height of approximately 30 cm. For the drought treatment, plants were treated with water deficiency. When the stress time was 0 d and 14 d, respectively, the 3rd to 5th leaves from the top of the plant were collected, weighed, and quickly placed in liquid nitrogen for freezing and preservation. For the salt stress treatment, 150 mL of 200 mmol/L NaCl solution was poured into the potted plants. when the stress time was 0 h and 24 h, the 3rd to 5th leaves from the top of the plant were collected, weighed, and quickly placed in liquid nitrogen for freezing and storage. For the RWC experiment, the third through fifth intact leaves from the top to the bottom of the plant were collected before and immediately after the drought and NaCl treatments, respectively, and the fresh weight of the collected leaves was measured. The leaves were placed in distilled water and the leaves saturated with absorbed water were weighed to obtain the saturated weight values, followed by drying in an oven at 105 °C for 6–8 h to obtain the dry weight values, and the results were calculated using the formula RWC (%) = [(Fresh weight) − (Dry weight)/(Saturated weight) − (Dry weight)] × 100%. POD activity was determined by the guaiacol method described by Maehly and Chance [58]. SOD activity of all extracts was determined photochemically using an assay system consisting of methionine, riboflavin, and NBT [59]. CAT activity was determined using the guaiacol method described by Aebi [60]. MDA content was determined by the thiobarbituric acid (TBA) method described by Heath and Packer [61], and Pro content was determined by the acid ninhydrin method of Bate et al. [62]; in the above experiments, three plants were selected from each line, and each plant was subjected to three biological replications and three technical replicates.

### 4.8. Yeast Two-Hybrid Assay

The pGBKT7-StMAPKK5 recombinant vector was constructed by homologous recombination method using *Nde* I and *Not* I as the restriction sites. After excluding that the pGBKT7-StMAPKK5 bait vector did not have self-activating activity and that the StMAPKK5 itself did not have any effect on the yeast two-hybrids, the potato cDNA library was co-transformed into the yeast receptor AH109 (Angyubio, Shanghai, China) with pGBKT7-StMAPKK5 by the PEG/LiAc-mediated method. After incubation on SD-TL selective medium at 30 °C for 5 d, colonies larger than 2 mm were picked and diluted in 10 μL of 0.9% NaCl solution, and 3 μL of the bacterial solution was spotted in SD-TLHA-x-α-gal (SD-tryptophan -leucine -histidine-adenine, SD-TLHA-x-α-gal) at 30 °C for 4–5 d. Colonies that turned blue were sent for testing, and the resulting sequencing results were analyzed [63].

### 4.9. Bimolecular Fluorescence Complementation Assay

To verify the reliability of Y2H, the CDS region of *StMAPKK5* without terminator was amplified into pSPYCE-35S vector using *Bam*H I and *Sma* I as the restriction sites, and the full-length coding sequences of the terminator-less *StMYB19*, *StZFP8*, *StPUB-like*, and *StSKIP19* were ligated into pSPYNE-35S to obtain the recombinant plasmid and then transformed it into *Agrobacterium* GV3101(Angyubio, Shanghai, China). The primer sequences are shown in Supplementary Table S2. Using pSPYCE-StMAPKK5 and pSPYNE-35S as the negative control, the bacterial fluids containing pSPYCE-StMAPKK5 and pSPYNE-StMYB19/StZFP8/StPUB-like/StSKIP19 were mixed in a 1:1 ratio and injected into tobacco leaves, and the methods of injection and infection were referred to the subcellular localization. After 48–72 h, the expression site of yellow fluorescent (YFP) protein was observed by a laser confocal scanning electron microscope (CARI ZEISS, LSCM800, Zeiss, Oberkochen, Baden-Württemberg, Germany) [64].

## 5. Conclusions

In this study, *StMAPKK5* was cloned from the potato variety ‘Atlantic’, which has a full-length sequence of 3648 bp, a full-length CDS of 1548 bp, and encodes 515 amino acids. *StMAPKK5* was expressed in roots, stems, leaves, tubers, and tuber buds with the highest expression in leaves. Subcellular localization showed that *StMAPKK5* was localized in the nucleus, cytoplasm, and cell membrane. *StMAPKK5* overexpression positively regulated and enhanced the drought and salt tolerance of the potato plants. Under drought and salt stress, the transgenic *StMAPKK5* plants showed increased activities of CAT, POD, SOD, and Proline in the antioxidant system, and reduced MDA content. The interaction proteins showed that StMAPKK5 interacted with StMYB19, StZFP8, StPUB-like, and StSKIP19. The expression analysis indicated that the interaction protein not only played a key role in the MAPKK signaling pathway, but also participated in other signaling pathways, and also played a significant function in drought and salt tolerance of potato crop. These results can provide a theoretical basis for further analyses of the functional analysis of the potato *StMAPKK5* and its possible mechanisms and signaling pathways under drought and salt stress conditions.

## Data Availability

The original contributions presented in the study are included in the article/Supplementary Material, further inquiries can be directed to the corresponding author/s.

## Supplementary Materials

The following supporting information can be downloaded at: https://www.mdpi.com/article/10.3390/ijms25073662/s1.
