# Supplementary material for: StMAPKK5 Positively Regulates Response to Drought and Salt Stress in Potato
Source: Int J Mol Sci. 2024 Mar 25;25(7):3662. doi: 10.3390/ijms25073662 (PMC11011605; doi:10.3390/ijms25073662)
Supplement: Supplementary file 1 [file ijms-25-03662-s001.zip › ijms-2893631-supplementary.pdf]

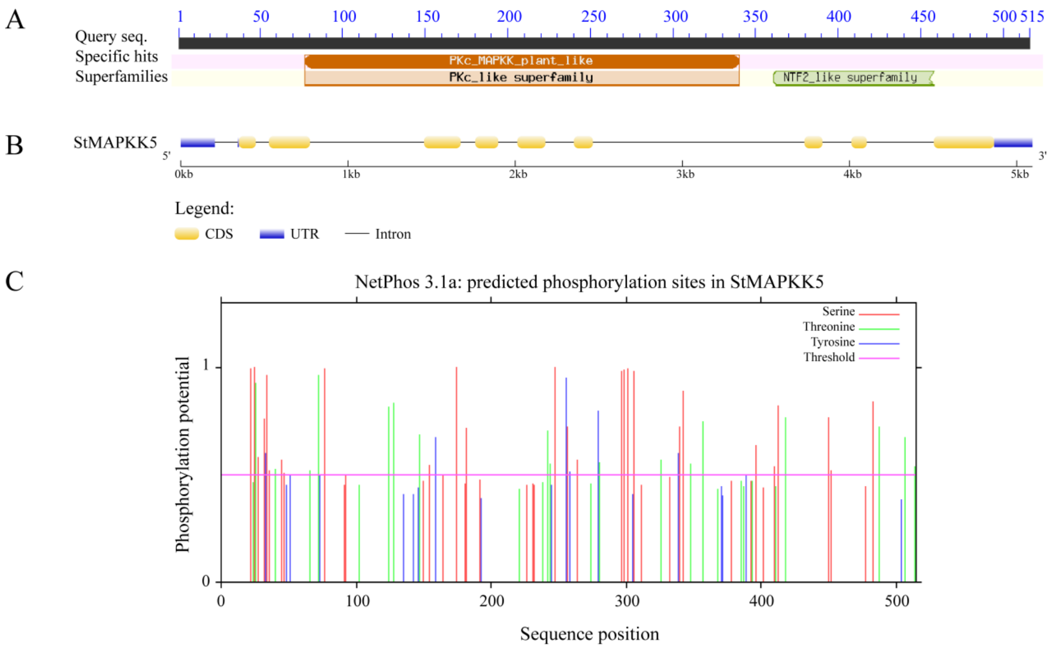

Supplementary Figure S1. Bioinformatics analysis of *StMAPKK5*. (**A**) Schematic diagram of the StMAPKK5 protein domain. (**B**) Schematic diagram of the gene structure of *StMAPKK5*. (**C**) Phosphorylation site of StMAPKK5 protein.

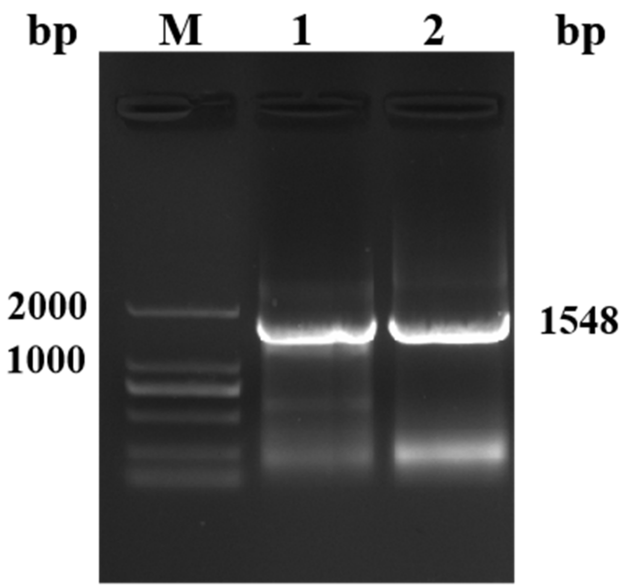

Supplementary Figure S2. Result of PCR product of *StMAPKK5* gene
M：DNA Marker DL2000; 1-2：*StMAPKK5* gene target segment

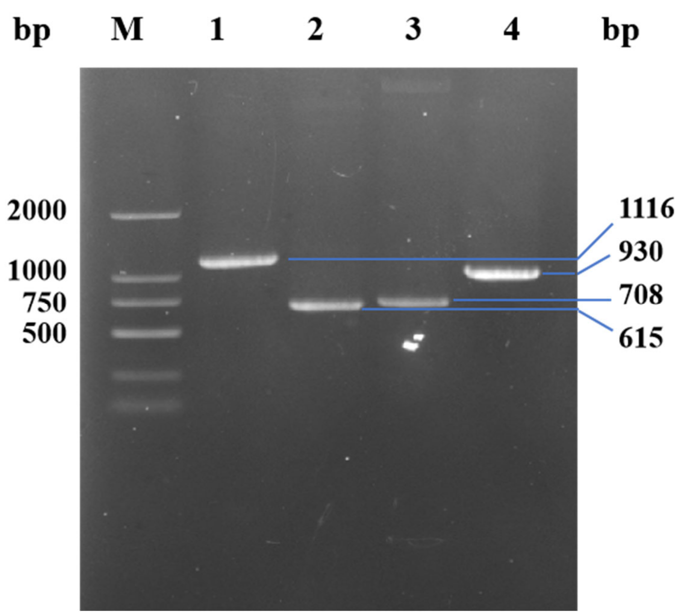

Supplementary Figure S3. Cloning of *StMYB19*, *StZEP8*, *StPUB-like*, *StSKIP19* genes
M：DNA Marker DL2000; 1:*StMYB* gene, 1116 bp; 2:*StZEP* gene, 930 bp;3:*StPUB-like* gene, 708 bp; 4:*StSKIP19* gene, 615 bp.

Supplementary Table S1. CDS sequences of the genes in this study

Gene	Gene ID	Coding sequence(CDS)
StMAPKK5（1548bp）	XM_006351467.2	ATGGCTGGACTGGAGGAATTGAAGAAGAAACTTGTGCCTTTGT<br>TTGATGCTGACAAGGGTTTTTCACCTACCTCTACATCCGATCCT<br>TTTGATTCATACTCTCTATCGGATGCCGGAACTGTGAATTTGTT<br>GAGTCAATCATATGGAGTATACAATATCAATGAGCTGGGATTAC<br>AAAAGTGGCCTGTTGATGACACGGATCATGGTGAAAAGACAT<br>ATAGATGTGCTTCCCATGAGATGAGGGTCTTTGGTGCCATAGGT<br>GCCGGTGCCAGTAGTGTTGTTCAGAGAGCAATTCATATTCCAA<br>CTCATAGGATTATTGCTTTGAAGAAGATAAATATTTTTGAGAAG<br>GAGAAAAGGCAGCAACTACTTACTGAAATAAGGACATTGTGT<br>GAGGCACCATGTTACCAAGGTCTTGTCGAGTTCTATGGAGCTT<br>TTTATACTCCTGATTCTGGGCAGATAAGCATAGCTCTAGAGTAC<br>ATGGATGGGGGTTCTCTTGCGGATATCATAAAAATTAGGAAGA<br>GCATTCCAGAACCTATCCTTTCCTCAATGGTTCAAAAGCTCTTG<br>CATGGTCTCAGTTATTTACACGGAGTTCGACATTTAGTCCACAG<br>AGACATAAAGCCAGCAAATTTACTTGTCAATCTCAAGGGGGA<br>GCCCAAAATAACTGATTTTGGAATTAGTGCTGGTTTAGAAAGC<br>TCAATTGCTATGTGTGCTACTTTTGTTGGAACAGTAACTTACAT<br>GTCGCCGGAACGAATACGGAATGAGAATTACTCTTATCCAGCT<br>GACATTTGGAGCCTTGGGCTTGCACTGTTTGAGTGTGGTACAG<br>GTGAATTTCCATATACAGCTAATGAAGGACCTGTCAATCTCATG<br>TTGCAGATCCTAGATGATCCGTCTCCTTCACTGTCAAGACACG<br>ACTATTCACCAGAATTCTGCTCATTCGTTGATGCTTGCCTCAAA<br>AAAAATCCTGATGACAGGCCGACAGCAGATCAGCTACTGTCA<br>CATCCATTCATTATCAAGTATAGTGATTCTGCATTAGACTTGGGT<br>ACTTTTGTCAGAGACATTTTTGATCCAACACAGAGGATGAAGG<br>ATTTGGCAGATATGCTGACGATACATTATTATTTACTATTTGATG<br>GATCCAATGAATTTTGGCAGCATACCAAGACATTATATAATGAA<br>TGCTCCACTTTCAGTTTTGGCGGGAAAGAATCCATCGGTCCCA<br>ACAATATTTTTTCAACCTTGTCCAACATACGGAACACATTAGCT<br>GGAGAATGGCCTCCAGAAAAGCTTGTCCATGTTGTAGAGAAA<br>CTTCAATGTCGAGCTAATGGTCAGGACGGAGTAGCAATTCGTG<br>TCTCTGGATCTTTTATAGTTGGAAATCAATTCCTCATTTGTGGAG<br>ATGGTATGCAAGTTGAGGGTCTGCCAAACTTGAAAGATCTCTC<br>TATTGATATACCCAGCAAACGGATGGGAACCTTTCATGAGCAG<br>TTTATTGTAGAGAAAGCAAACATTATCGGTCGTTATTTCATAAC<br>TAAGCATGAACTTTTCATTACTCAATAG
StMYB19（1116bp）	NM_001318681	ATGGCGAGTTCTAAGAGAGATATGGATCGGGTTAAAGGTCCAT<br>GGAGCCCTGAAGAAGATGAGCTTTTACAGCAACTTGTTCAGA<br>AACATGGCCCCAGAAATTGGTCTCTAATAAGCAAATCGATTCC<br>TGGGAGATCCGGCAAATCTTGCCGGTTGCGGTGGTGTAATCAG

		TTATCTCCACAGGTGGAGCATCGGGCTTTTACGCCTGAAGAAG<br>ATGAGACTATTATTCGGGCCCATGCCCGATTCGGTAACAAATG<br>GGCTACTATAGCCCGACTTCTTAATGGGAGGACTGATAACGCT<br>ATTAAGAATCACTGGAACTCTACCTTGAAGAGGAAGTGTTCAT<br>CGCTTAGTGCCGATGAAGGGAACGAACTCGCCGATCAAATTTT<br>TGAAAATCAGCAGCCGCCGTTGAAGAGATCTGTTAGTGCCGG<br>ATCCGCTATGCCGGTTTCGGGTTTTCATTTCAGTCCAGGTAGCC<br>CATCGGGTTCGGATAGTGATTCGAGTCTTCATGTTACGTCGTCG<br>TCTCAATCTCATGTGTTTAAGCCTGTTGCTAGAACCGGCGGCGT<br>ATTCCCGCAGTCGATCGATATCTCATCTCCGCCTGTTGATCCTC<br>CCACCTCCCTAAGCCTTTCGCTTCCTGGTGTTGACTTGGCTGAG<br>TTCTCTAATCGTTCGGCCGATTCGACTCAGTCGAAAAATCCTTT<br>TCAGTTGCTGCTTCCACCGATGCAGATTCCCCCACCGCCGCCA<br>CCACCACCACCACCGCCGCCGCAGGCAACGACGGTACCTTTC<br>GAACGAGTTTCTGCAATTCAACAGTCTCTTCAGAACCCTGATT<br>TCGGGAAGAACGCAGGAGGAGAACAGCCAGACAAGGTATTT<br>GTGCCGTTTAGCCAGGAGTTGTTAGGGGTGATGCAGGAGATGA<br>TAAAGACGGAGGTGAGGAACTATATGATGGGAGTTGAACAAA<br>AGCAGCAGTTTCAGCAGCAGCAGCAGCATTATCAACAACAAC<br>CGCAGCAGTTTCAGCAACAAAACCATCAATTGCCAAGTGGTTT<br>AGGGCTTGGATTGTGTATGCAACAAGCTTCTGATGGATTCAGG<br>GATAGAGCTGCTAATCGCATGAGACTAAGCAAATTCGATTAG
StZFP8（615bp）	XM_006362556.1	ATGCCTCATCAGAATCAGGGACAAAATGGGCAGCAGGGTGTG<br>TGGATGTACCAATGCATCCATTGTAAAAAAAACTTCCCTTCAA<br>GCCAGGCCATAGCAGGCCATACAAAGGGTCATTTCAGAGATG<br>GCTGGGTTAAAGGAACCCCCCAGAGCAAAGTATTTGTACTATT<br>TTCTGAGTATCAGCAGCAGCAGGGTTCTATTACTGACTCTTCAA<br>TTCCCGAGAAACATGTTTTTTCTGCAGCAACATCTGACACTGA<br>CTTAACAGATGCTCATCGATTGCCAATAGGTGATGTGCAGAATT<br>CCAGAAGCCTGGTCGTACGGCCTCCATCTCCAGCTTCGTCTAG<br>CAGACAACCACGAATCCCTAGGCATCATTTGAGGCTTCGTGAT<br>CTGAAGATACTAGCTAGGCTAAGGGCTCGTCTTACCAGAGAGG<br>AGCAAGAAGTCATTTTGCGTCTTCTTGATAGTGCTATGGAACA<br>GGCAAAACAATCAAGCAAGAAGAGCACTGAGGCGGAGGTGA<br>TCCCAAATAGAAACTCAGAAGCAGCAGCCGCGATTGGAACTA<br>CTGACACAGATACCGATGTCTCTTCTGAGGAATCAGATGATGA<br>GTCCAAAAATATGTGA
StPUB-like（708bp）	XM_015304533.1	ATGGGTGCAGTTTGTTGCTGCTTGCGAGACGAATGTGAAGATT<br>TTGCCAACCCAAACAGCTCAATGTATAGGAACTGTATATGCTTT<br>CCAACTCTTGTTCAGAACTTCTTGCATGTGTATGCATCACTTTT<br>CCATAGAGGAGAAAGACATGTCATTCCTTCATCGGATCAAGGT<br>GCAGCATCTTTGAGTTCTACAGCATCTCTTGATGACTCACTATC<br>TGACATGTACCGTTCTCCTCCGAGACCACTGCCATATGATGCTG<br>ATCCAAGATATTTCCGCTTGCAACAAGATGGACAAGTCCCAAG

		AAGGGAGAAAGGCTCGAGCCACTCATATGAGGAAACTGAAC<br>CACTACAAAGAAATTTTGATGACCCTGAATCGTTAAGTGATGT<br>AAATAAATGGAGTCTGCCTACATTTGAAGAAGGATCAAAAGA<br>ATACAATAAGTCTTCTGTGGAATTCTCAACTGCTAAAATGACTT<br>CTGGAGATGCTCATAGCTATTATTATTCAGAAGATGAAGATGTC<br>TGCCCAACCTGTCTTGAAGAATATACAGAGGAAAACCCAAAA<br>ATAATAACAAAATGCTCTCACCATTTCCACCTGAGTTGTATATA<br>TGAGTGGATGGAAAGAAGTGACAGCTGTCCAGTTTGTGGCAA<br>GGTAATTGTGATATCATTTAAATTTATGTGTCATCTCCTTCTCTTC<br>TTTTTTAATGCTTGA
StSKIP19（930bp）	XM_015303010.1	ATGGAAGCAGAAGATATGGAAAATTCAATTGCTCCGCCGTGG<br>GTGGAATTGCCGCCGGATATTACATCGTCGATCCTTCAAAAGG<br>TTGGGCCAATTGAGATGTTGAAGAGCGCCGACAAAGTGTGTTC<br>AACTTGGCGTCGGTTGTGCCATGAACCAGCTATGTGGCGAGTT<br>GTGAAAATGCAGAATGCCGGCGCCGATTTCTGGGACAGGGAG<br>GATGATTTGGAGAAGATTTGCGGTCGAGCTGTGGAATGTAGCA<br>ACGGGGAATTAGTTGATCTCAGCCTTGAGTATTTTGGCAGCGA<br>CAAGTTGCTCAGCTATATCTCTGAAAGATCACCTCAGCTCAAA<br>CGTCTTCGACTCATATGTTCCTACAATGTCTCAGCCGAGGGATT<br>GAGTGCAGCTGTGAAGAAGTTCCCATTATTGGAGGAGCTGCA<br>CCTCTATTACATCGCTATCACCAAAGAAGCGATTGAAACTATA<br>GGCAGTTCTTGTCGTGGTTTGAAGTCTTTTAAGTTGAACAACC<br>AGATCTGCAGACACCCCTACATTGAATACGATGAAGAAGCCAT<br>AGCTATTGCACAGAACATGCCTGAACTGCACCACCTTCAACTC<br>CTTGGAAACAAGATGACGAATGAAGGATTGGAAGCTATTCTC<br>AATGGCTGCCCTCAACTTGAATCGCTTGATCTGCGGCGATGTCT<br>CAATGTCGATCTGGGAGGGGAAGTAGGTAAGCGATGTTCTCGA<br>CAGATCAAATCTCTAAGACACCCTCAGGACCCCACCGAAGATT<br>ATGGGCTTGATACAGAGATGCACGACTTTGAGTCGTTTGATGA<br>GGATTACCCATCTGGATTTTCTGACATTGACCTCATATCAGACG<br>ACGATGATGATTATGAGTTTTCTGATGGCAGCAACTATTCTGAT<br>GATGATGAGATGGTGTTCGAATATTGA

Supplementary Table S2 Primer information used in this study

PCR primers for the recombinant plasmid		
pCAMBIA1300-35S-StMAPKK5	Forward	5′-CGGGGGACGAGCTCGGTACCATGGCTGGACTGGAGGAA-3′
	Reverse	5′-TGCTCACCATGTCGACCTATTGAGTAATGAAAAGTTCA-3′
pCAMBIA1300-EGFP-StMAPKK5	Forward	5′-CGGGGGACGAGCTCGGTACCATGGCTGGACTGGAGGAA-3′
	Reverse	5′-TGCTCACCATGTCGACTTGAGTAATGAAAAGTTCA-3′
pCPB121-aimRNA-StMAPKK5	amiRNA	5′-TATATCCGCAAGAGAACGCCC-3′
	I miR-s	5′- GATATATCCGCAAGAGAACGCCCTCTCTCTTTTGTATTCC-3′
	II miR-a	5′-GAGGGCGTTCTCTTGCGGATATATCAAAGAGAATCAATGA-3′
	III miR*s	5′-GAGGACGTTCTCTTGGGGATATTTCACAGGTCGTGATATG-3′
	IV miR*a	5′-GAAATATCCCCAAGAGAACGTCCTCTACATATATATTCCT-3″
	A	5′-CTGCAAGGCGATTAAGTTGGGTAAC-3′
	B	5′- CTGTTTCCTGTGTGAAATTGTTATCCGC -3′
pGBKT7-StMAPKK5	Forward	5′-GGAGGACCTGCATATGATGGCTGGACTGGAGGAA-3′
	Reverse	5′-GCTAGTTATGCGGCCGCCTATTGAGTAATGAAAAGTTCA-3′
pGADT7-StMYB19	Forward	5′-AGGAGGACCTGCATATGGCGAGTTCTAAGAGAGAT-3′
	Reverse	5′-GCTAGTTATGCGGCCGCTTTTTTTTTTTTTTTTTTTTCAAT-3′
pGADT7-StZEP8	Forward	5′-AGGAGGACCTGCATATGATGCAGCAACAACAACCATCA-3′
	Reverse	5′-GCTAGTTATGCGGCCGCTTCCTTCAGAATAGCAAAACATACA-3′
pGADT7-StPUB-like	Forward	5′-AGGAGGACCTGCATATGTTTTCCCATCTTTTACTTTGTTATC-3′
	Reverse	5′-GCTAGTTATGCGGCCGCTTAAAGTGAAAACCATATAACAAT-3′
pGADT7-StSKIP19	Forward	5′-AGGAGGACCTGCATATGAATTAGGGCAAATTAACTCAAAAT-3′

	Reverse	5′-GCTAGTTATGCGGCCGCTGATTCGAATGTCCTTCATAAAAGT-3′
pSPYCE-StMAPKK5	Forward	5′-CGCCACTAGTGGATCCATGGCTGGACTGGAGGAA-3′
	Reverse	5′-GTATGGGTACATCCCGGGTTGAGTAATGAAAAGTTCA-3′
pSPYNE-StMYB19	Forward	5′-CGCCACTAGTGGATCCATGGCGAGTTCTAAGAGAGATATGG-3′
	Reverse	5′-CTTTTGCTCCATCCCGGGATCGAATTTGCTTAGTCTCATGCG-3′
pSPYNE- StZEP8	Forward	5′-CGCCACTAGTGGATCCATGCCTCATCAGAATCAGGGA-3′
	Reverse	5′-CTTTTGCTCCATCCCGGGCATATTTTTGGACTCATCATCTGA-3′
pSPYNE-StPUB-like	Forward	5′-CGCCACTAGTGGATCCATGGGTGCAGTTTGTTGCTG-3′
	Reverse	5′-CTTTTGCTCCATCCCGGGAGCATTAAAAAAGAAGAGAAGGAGA-3′
pSPYNE-StSKIP19	Forward	5′-CGCCACTAGTGGATCCATGGAAGCAGAAGATATGGAAAAT-3′
	Reverse	5′-CTTTTGCTCCATCCCGGGATATTCGAACACCATCTCATCATCA-3′
qRT-PCR primers for the plant		
StMAPKK5	Forward	5′-CTTACATGTCGCCGGAACGA -3′
	Reverse	5′- CAAACAGTGCAAGCCCAAGG -3′
StEf1α	Forward	5′-CAAGGATGACCCAGCCAAG-3′
	Reverse	5′-TTCCTTACCTGAACGCCTGT-3′
StMYB19	Forward	5′-TTTGAAAATCAGCAGCCGCC-3′
	Reverse	5′-CGAAAGGCTTAGGGAGGTGG-3′
PUB-like	Forward	5′-TTTGTTGCTGCTTGCGAGAC-3′
	Reverse	5′-ATGCTGCACCTTGATCCGAT-3′
StSKIP19	Forward	5′-GTGGAATGTAGCAACGGGGA-3′
	Reverse	5′-ACAGCTGCACTCAATCCCTC-3′
StZFP8	Forward	5′-AGCAGGCCATACAAAGGGTC-3′
	Reverse	5′-GACGAAGCTGGAGATGGAGG-3′
PCR primers for the transgenic plant		
NPT II	Forward	5′-GCTATGACTGGGCACAACAG-3′
	Reverse	5′-ATACCGTAAAGCACGAGGAA-3′

HYG	Forward	5′-GCTTCTGCGGGCGATTTGTGT-3′
	Reverse	5′-GGTCGCGGAGGCTATGGATGC-3′
